# Automatic Detection of Cracks on Concrete Surfaces in the Presence of Shadows

**DOI:** 10.3390/s22103662

**Published:** 2022-05-11

**Authors:** Paulius Palevičius, Mayur Pal, Mantas Landauskas, Ugnė Orinaitė, Inga Timofejeva, Minvydas Ragulskis

**Affiliations:** Centre for Nonlinear Systems, Department of Mathematical Modelling, Kaunas University of Technology, 51368 Kaunas, Lithuania; paulius.palevicius@ktu.lt (P.P.); mayur.pal@ktu.lt (M.P.); mantas.landauskas@ktu.lt (M.L.); ugne.orinaite@ktu.lt (U.O.); inga.timofejeva@ktu.lt (I.T.)

**Keywords:** concrete crack detection, deep learning, convolution neural networks, image classification, image augmentation

## Abstract

Deep learning-based methods, especially convolutional neural networks, have been developed to automatically process the images of concrete surfaces for crack identification tasks. Although deep learning-based methods claim very high accuracy, they often ignore the complexity of the image collection process. Real-world images are often impacted by complex illumination conditions, shadows, the randomness of crack shapes and sizes, blemishes, and concrete spall. Published literature and available shadow databases are oriented towards images taken in laboratory conditions. In this paper, we explore the complexity of image classification for concrete crack detection in the presence of demanding illumination conditions. Challenges associated with the application of deep learning-based methods for detecting concrete cracks in the presence of shadows are elaborated on in this paper. Novel shadow augmentation techniques are developed to increase the accuracy of automatic detection of concrete cracks.

## 1. Introduction

Concrete structures like bridges, beams, columns, and highways are often subjected to high levels of stress and strain. The stress in the concrete structures is caused by continuous cyclic loading, changes in temperature, and effects of weathering, which could result in the origination and propagation of cracks in concrete structures. Sometimes these cracks become connected and can increase in size [1,2]. Early detection of a failure in concrete structures in offshore and onshore environments, bridges, concrete pillars, and concrete pipelines helps to put preventive measures in place to avoid failures, which can save assets and lives.

Crack detection is done by using invasive or non-invasive techniques. Invasive techniques, which usually involve surveying using specialized equipment, like infrared light, thermal testing, ultrasonic techniques, and testing concrete samples in the laboratory, are often time-consuming and complex processes. All the invasive and non-invasive methods require structural experts to analyze and interpret the available data [3]. The findings of such methodologies are often subjected to human interpretation and knowledge. With advent of improved imaging capabilities and increased computational power, other non-invasive techniques using digital image analysis of concrete structures have gained a lot of momentum. In the last few decades, more than 50 articles discussed the problems of concrete crack identification using pre-processing and post-processing techniques. A comprehensive overview of such methods (including advantages and disadvantages) are presented by Mohan et al. [4].

More recently, deep learning-based methods using ANNs (artificial neural networks) and CNNs (convolutional neural networks) have been applied to automatically process the images for crack/failure identification in onshore concrete installations [5,6,7,8,9]. Several applications based on the use of CNN-type neural networks to identify surface concrete cracks have recently emerged with the development of artificial intelligence and deep learning technologies. Many of these methods could be characterized by high classification accuracy. Most of the published data spans the last decade. Kim et al. [5] presented a conference paper in 2011 in which they used a backpropagation neural network for 225 concrete images. The network was trained using 105 images, and the trained network was tested for 120 new images. The recognition rate of the crack image was 90% and non-crack image was 92%. Choudhary et al. [10] published a methodology for crack identification and detection using an object detection method. In their work, they have utilized over 205 256 × 256 resolution images, claiming a high crack detection accuracy reaching 96%. Ha [11] published their work in 2016 in which they have used an image segmentation method for automatic peak detection enabling concrete crack identification.

The first extensive data set of over 40,000 images of 256 × 256 resolution is used by Cha et al. [6] in their 2017 paper. They have applied a CNN-based deep learning method for concrete crack detection achieving very high accuracy of over 98%. A series of papers published in 2019 using deep learning methods for crack identification and detection. Papers published by Chen et al. [12] and Cao et al. [13] use CNN-based methods on the same set of 40,000 images of 227 × 227 resolution, both presenting good recognition accuracy of over 99% and 90%, respectively. The 2019 paper published by Lee et al. is distinguishable as they have used very high resolution (3120 × 4160) data set comprising 60,000 images for training and testing of their CNN-based crack identification method with a very high prediction accuracy of about 99%. A series of papers published in 2019 by Moon et al. [14] and Kim et al. [15] also use convolution neural networks.

More recently, Kim et al. [5] have used the same data set [6,13] and have further improved the accuracy of the CNN to 99.9%. Jitendra et al. [16] and Wenming et al. [13] have presented a comparison of different deep learning networks in their papers published in 2020. They have also used over 20,000 images of 1024 × 1024 higher resolution images. Long-short term memory (LSTM) based deep learning convolutional neural networks have also been applied for crack identification [17]. This paper is unique in the sense that the use of the CNN-LSTM type method has not been presented before in literature for problems of crack identification.

A recent paper was published in 2021 where thermal image-based crack identification and detection is performed using a U-net type learning network [8]. However, the prediction accuracy of the U-net based on thermal images is rather low and reaches only 78%. In 2021, a paper presented by Yang et al. [18] shows a comparison of three neural networks, Alexnet, VGGNet13, and ResNet18, to recognize and classify crack images. This paper also shows that the trained YOLOv3 model detects the crack area with a satisfactory accuracy.

Although all these methods claim very high accuracy, they often ignore the complexity of the image collection process itself. Almost all published papers deal with images captured in ideal laboratory-based conditions. None of the published papers have specifically considered the challenge of identifying concrete cracks in the presence of shadows. One possible approach for dealing with this problem is shadow detection and its removal. However, shadow removal is far from being a straightforward task. Early papers on shadow detection and removal have been presented by Finlayson et al. [19,20]. A comprehensive detail of the various shadow detection and removal methods is presented by Murali et al. [21]. More recently, ANN-based deep learning methods have also been deployed for shadow detection and removal [22,23,24]. Although several researchers have tried to solve the problem of shadow removal; this task still remains a complex topic with moderate to good success [21]. Shadow removal in concrete crack images often severely impacts the quality of the digital image, which makes further crack detection very challenging [25].

A new approach for realistic crack detection using the augmentation of existing crack data sets by complex shadow shapes is proposed in this paper. The presented methodology helps to automate the crack detection in real environmental settings. Moreover, it enables the use of the deep learning-based crack identification methodology for broader real-world applications, including the use of camera-carrying areal unmanned vehicles.

This paper is organized as follows: Section 2 presents concrete crack detection challenges in the presence of shadows and the proposed concrete crack identification framework. Results are presented in Section 3. Section 4 presents discussions. Finally, conclusions follow in Section 2.2.

## 2. Materials and Methods

This section, first, presents the details of challenges associated with crack detection using image analysis. Second, details of the methodology proposed in this paper to help improve crack detection with improved image classification algorithms follow next. Finally, the implementation of the algorithm is presented.

### 2.1. Concrete Crack Detection Challenges in the Presence of Shadows

**Figure 1:**
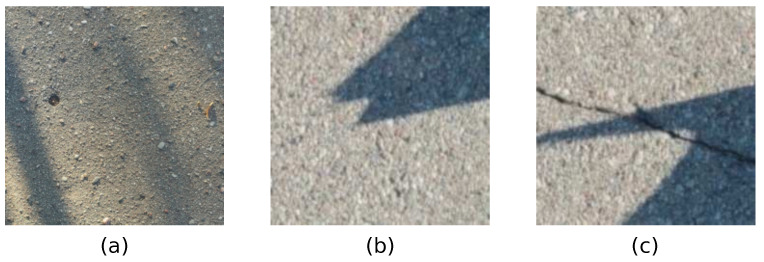
Sample images of concrete surfaces in the presence of shadows. Parts (**a**,**b**) show concrete surfaces without cracks. Part (**c**) shows concrete crack surface with crack.

Crack identification and characterization in images containing concrete cracks is a demanding task. Images of concrete surfaces containing cracks taken in real-life conditions are impacted by complex illumination conditions, including shadows and shading. An example of concrete images containing cracks and shadows is shown in Figure 1. Current state-of-the-art image analysis methodologies that are applied to real-world images of concrete structures in the presence of shadows can result in misleading results, which is further discussed and shown in this Section.

**Figure 2:**
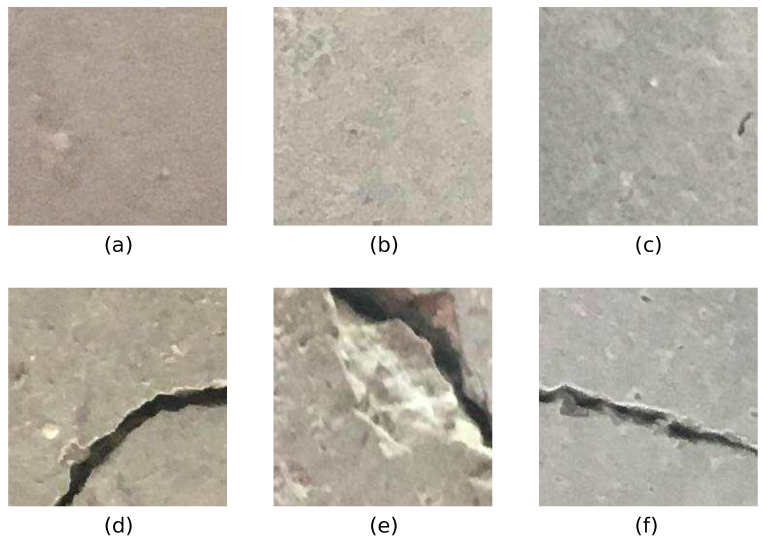
Some typical images from the Mendeley’s data set of “Concrete crack images for classification” [26]. Shadows are absent in all images. Parts (**a**–**c**) show concrete surfaces without cracks. Parts (**d**–**f**) show concrete surfaces with cracks.

Some sample images are used to demonstrate crack detection challenges in the presence of shadows using existing deep-learning-based methods. For this purpose, we will use the published data set of concrete crack images from Ozgenel [26], which consists of 40,000 images of surfaces with and without concrete cracks. A set of sample images is shown in Figure 2. This database of images has been widely used by researchers for the training and testing of multiple deep learning models for concrete crack detection.

**Figure 3:**
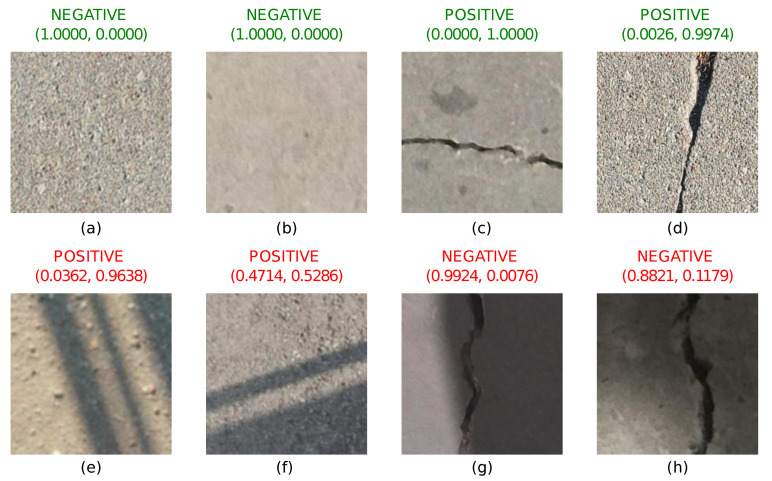
The model trained on the original Mendeley “Concrete Crack Images for Classification” produces wrong results in the presence of shadows. Images without cracks and shadows in parts (**a**,**b**) and with cracks and without shadows in parts (**c**,**d**) are classified correctly. Images without cracks but with shadows in parts (**e**,**f**) and images with cracks and shadows in parts (**g**,**h**) are classified incorrectly. Figure highlights the deficiencies of the standard image classification algorithms.

The test case based on the paper by Byunghyun et al. [27] (which uses a classification network AlexNet [28]) is used in the following experiments. The network is trained on a subset of “Positive” and “Negative” images of 227 × 227 resolution and then tested on a subset of test images for accuracy. Testing results of sample images of different scenarios are shown in Figure 3. Images without shadows and without cracks are shown in Figure 3a,b. Model accuracy is perfect, as expected for this kind of image. The same accuracy is also achieved in Figure 3c,d. These images contain cracks, but shadows are absent. Images with shadows and without cracks are shown in Figure 3e,f. These images demonstrate False-Positive cases—shadow patterns are incorrectly identified as cracks. Finally, images with shadows and with cracks are shown in Figure 3g,h. It has been found that images having large shadow areas of high intensity can result in False-Negative errors. Thus, it is clear that a classification network trained on images captured in laboratory conditions without shadows can result in the wrong classification of images with shadows.

This test example shows the disadvantages of training deep learning models on ideal images of cracked concrete surfaces. It raises an important question of whether these models could be generalized for real-life images, which are often impacted by varying illumination conditions. The first approach that comes to mind would be to improve the classification and segmentation accuracy of concrete images containing shadows by applying shadow removal pre-processing techniques. However, shadow removal is not a straightforward task. It has been shown that pre-processing of concrete crack images for shadow removal could lead to severe deterioration in image quality, leading to incorrectly classified images [25].

### 2.2. The Concrete Crack Identification Framework

Examples presented in the previous section clearly demonstrate that the deep learning methods trained on images containing concrete surfaces without shadows cannot correctly classify concrete crack images in the presence of shadows. In order to overcome this challenge, it is necessary to re-train the network on large data sets of concrete crack images containing shadows. However, the creation of such a database is not an easy task as it requires diligent image collection of concrete surfaces with different shadow shapes. We propose a shadow augmentation technique that allows reusing existing data sets of concrete crack images. We also provide details on the deep learning image classification network, which is further used to test the accuracy of the model that is trained using augmented data.

### 2.3. The Proposed Shadow Augmentation Technique

**Figure 4:**
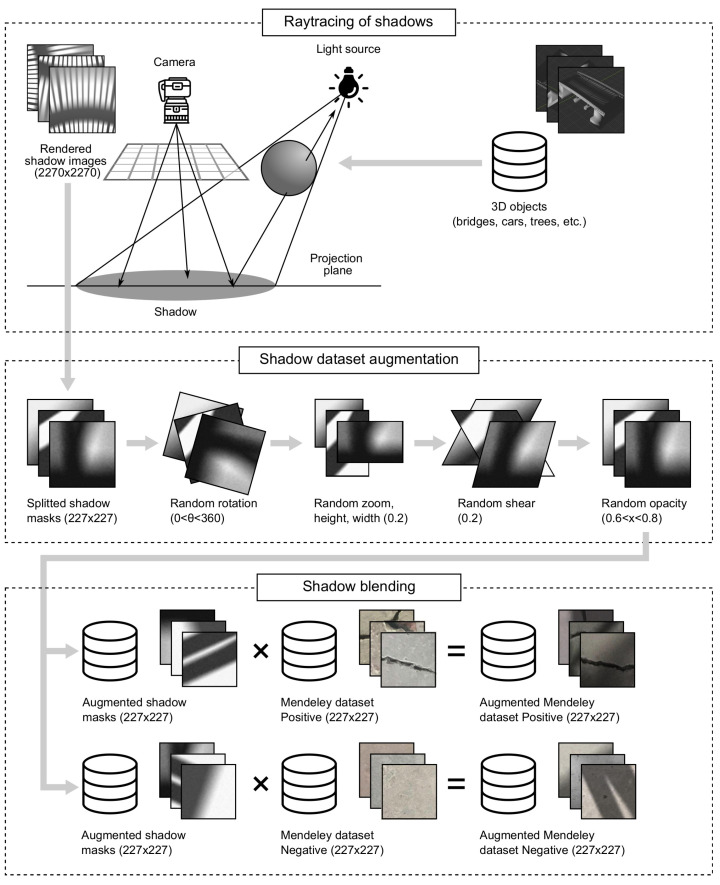
A schematic diagram for synthetic augmentation of complex shadow patterns—ray-tracing of shadows, shadow data set augmentation, and shadow blending.

We propose a three-step shadow augmentation technique: (1) ray-tracing of shadows, (2) augmentation of shadow masks, and (3) shadow blending. The whole augmentation process is depicted in Figure 4.

Firstly, the computational ray-tracing of shadows is performed in a virtual optical environment. A wide variety of realistic shadow images are generated using the 3D computer graphics software Blender with the Cycles rendering engine [29]. Cycles is an unbiased physically-based render engine that utilizes path tracing to represent real-world optical phenomena accurately. Path tracing is a type of ray tracing technique used to simulate the physical behavior of light. This family of algorithms is based on approximating the solution to the rendering equation, which describes the light propagation in a scene via the following integral equation [30]:

(1) $$L_{o}\left(x,\omega_{o}\right)=L_{E}\left(x,\omega_{o}\right)+\int_{\Omega }L_{i}\left(x,\omega_{i}\right)f_{r}\left(x,\omega_{i},\omega_{o}\right)\left(\omega_{i}\cdot n\right)d\omega_{i},$$

where x is the space variable, $\omega_{o}$ is the direction of the outgoing ray of light, $L_{o}$ is the total spectral radiance directed from the point *x* along the direction $\omega_{o}$, $L_{o}$ is emitted spectral radiance, Ω is the unit hemisphere in direction of normal vector n at x, $\omega_{i}$ is the direction of the incoming ray of light, $L_{i}$ is spectral radiance coming inward to x from the direction $\omega_{i}$ and $f_{r}\left(x,\omega_{i},\omega_{o}\right)$ is the bidirectional reflectance distribution function (the proportion of light reflected from the direction $\omega_{i}$ to $\omega_{o}$ at x).

As solving Equation (1) is a computationally extensive task, it is common to approximate the integral in Equation (1) by utilizing the Monte Carlo simulation as follows [30]:

(2) $$\int_{\Omega }f\left(x\right)d\Omega \approx \frac{1}{N}\sum_{X}f\left(x\right),$$

where f(x) is an arbitrary function and *X* is a set consisting of *N* samples uniformly distributed in Ω. In order to improve the convergence speed of this method, path tracing techniques apply a methodology based on deterministic sampling, called the Quasi-Monte Carlo method.

The short outline of the path-tracing algorithm used in the Cycles rendering engine is as follows: multiple rays are cast from each pixel of the camera into the scene in random directions. The produced rays reflect, refract, or get absorbed by the objects in the scene until they either reach a light source or the user-defined bounce limit, forming a set of paths from the camera to the light. The amount of light per pixel is then calculated for each ray, the value is averaged and assigned to that specific pixel. In our case, multiple 3D objects are used to render 50 images. The individual size of each rendered shadow image is 2270 × 2270, which is then automatically spitted into 100 different shadow masks. This has been demonstrated in the first block of Figure 4.

One approach would be to generate a separate shadow mask for each image in Mendeley Concrete Crack Images for Classification dataset containing 40,000 images. However, we propose using standard image transformation techniques to augment the shadow mask data set (in our case, this data set contains 5000 unique shadow masks). This is demonstrated in the second block of Figure 4. Transformations, such as random rotation (0<θ<360deg), random zoom (0.2), height (0.2), width (0.2), shear (0.2), and opacity (0.6<x<0.8) has been applied to generate 40,000 unique shadow masks. The parameter used for random rotation is between 0 and 360deg as shadows can be cast over concrete images at any angle. Other parameters were selected experimentally to prevent divergence from original shadow forms.

**Figure 5:**
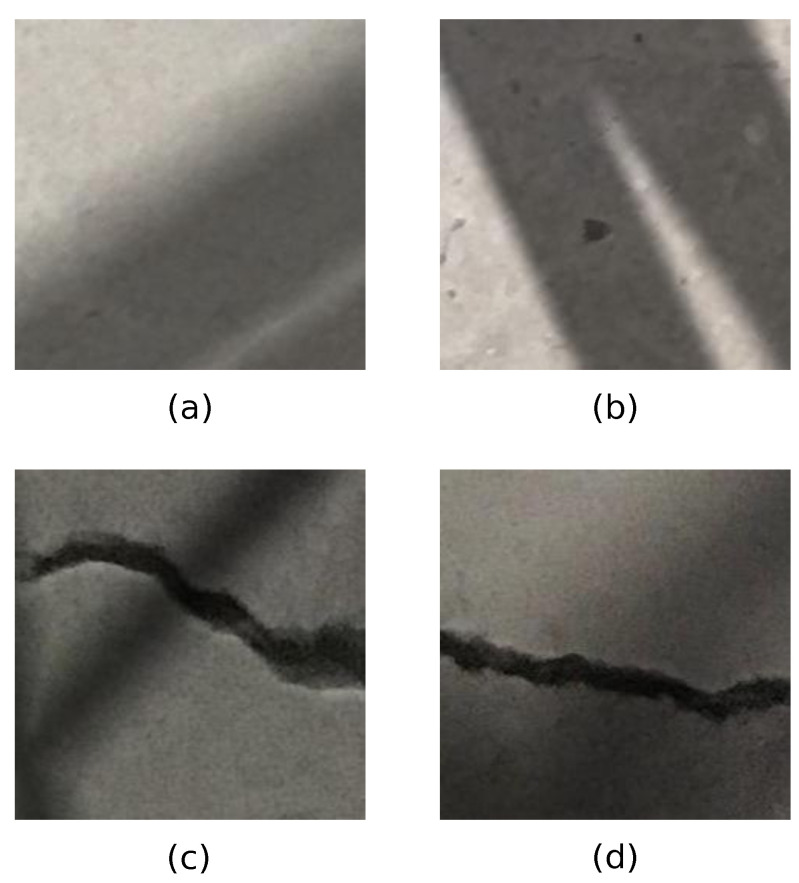
Typical images after the application of shadow augmentation technique for images in Mendeley Concrete Crack Images for Classification data set: (**a**,**b**) depicts images without cracks; (**c**,**d**) depicts images with cracks.

Finally, augmentation of concrete crack images is performed by image combination through superposition techniques, where one image (shadow mask) is blended with another image (concrete surface) to create an illusion of a single image containing features from both source images. This effect is achieved by using a multiply blending operation. Multiply blending takes values from 0 to 1 of each pixel in the first image and multiples them with the values for the corresponding pixel from the second image. Wherever either layer was brighter than black, the composite is darker because each value is less than 1. The product will be less than each initial value that was greater or equal to zero. This operation (as shown in Figure 4) is performed for the whole data set of 40,000 images (once for images with and once for images without cracks). Typical resulting images of the augmented Mendeley Concrete Crack Images for Classification data set are shown in Figure 5.

### 2.4. Neural Network for Concrete Crack Detection

In this section, we provide details and architecture of a deep learning image classification network AlexNet [28], which we use to train the classification model using augmented data. AlexNet is a convolutional neural network developed in 2012 by A. Krizhevsky et al. [28]. It was the winning entry in the ImageNet Large Scale Visual Recognition Challenge (ILSVRC 2012) which involved classifying images (227 × 227 pixels) into 1000 different classes (e.g., cats, dogs) [31]. AlexNet is a large neural network comprising 25 layers (input and output layers, 5 convolutional layers, 3 max-pooling layers, 3 fully connected layers, 7 ReLU layers, 2 normalization layers, 2 dropout layers, and 1 1000-way softmax layer), 60 million parameters and 650,000 neurons. Its architecture is given in Table 1.

Compared to previous neural networks, there were several advanced techniques used in AlexNet, which significantly enhanced its performance. Firstly, the rectified linear unit (ReLU) function (ReLU(x)=max(x,0)) was utilized in order to eliminate the gradient vanishing problem which often follows the traditional activation functions (for example the logistic function). As the gradient of ReLU is always equal to one when the input is larger or equal to zero, it has been shown that the convergence speed of deep neural networks with ReLU as the activation function is faster than traditional activation functions, which greatly accelerates the training procedure. Next, two-dimensional convolutional layers with trainable kernels were used to produce a feature map and max-pooling was utilized for feature reduction. Max-pooling is a common technique that considers a group of neighboring pixels in the feature map and computes their maximum value. Moreover, local response normalization was introduced to aid generalization [28]. The dropout technique was used to avoid overfitting and accelerate the training process. Dropout freezes neurons at random with a set dropout probability preventing their engagement in forward and backward passes during the training phase. Finally, a 1000-way Softmax activation was utilized to produce a distribution over the 1000 class labels.

As mentioned in the previous sections, the concrete image classification problem this paper analyzes involves only two classes—“with cracks” and “without cracks”. Thus, the last layer of the original AlexNet architecture (see Table 1) must be modified: 2-way Softmax activation needs to be used at the output. Due to the reduced number of outputs, the two fully connected layers right before the last layer may also be modified by reducing the number of neurons in them if severe overfitting or a very slow convergence is observed during the training of the network. Next, the learning rate factor for weights and biases as well as the initial learning rate are adjusted to slow down the learning speed of the new network and increase its prediction capabilities. Finally, the new network is retrained with the augmented data set of concrete images.

The training of the network using the augmented data set of concrete images was performed by using the stochastic gradient descent optimizer. The batch size was set to 15 samples of augmented concrete images. The number of training epochs was limited to 25. Training and testing samples comprised 85% and 15% of the augmented data set accordingly. The initial learning rate was fixed to 0.01. All these parameters were tuned after carrying out extensive numerical computations.

**Figure 6:**
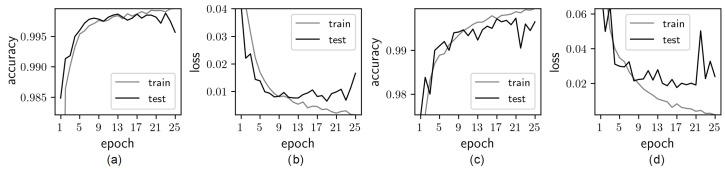
The training dynamics of the network: (**a**,**b**) depicts the accuracy and loss functions using the original data set; (**c**,**d**) correspond to the training dynamics of the network with the augmented data set.

The training dynamics are depicted in Figure 6. Note that the network starts to overfit after epoch 9 (although the optimization process still continues). This is true both for the original and the augmented data sets. Thus, it is possible to conclude that only 9 epochs are sufficient to train the network.

## 3. Results

**Figure 7:**
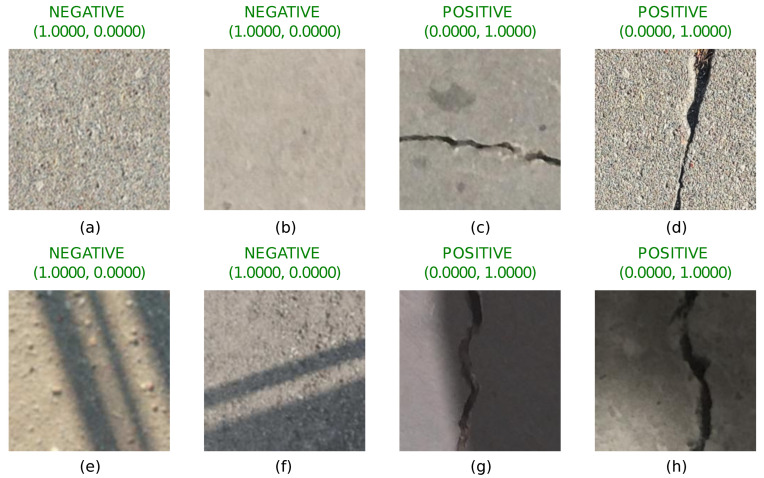
The model trained on the augmented Mendeley “Concrete Crack Images for Classification” produces correct results in the presence of shadows. Images without cracks and shadows in parts (**a**,**b**) and with cracks and without shadows in parts (**c**,**d**) are classified correctly. Images without cracks but with shadows in parts (**e**,**f**) and images with cracks and shadows in parts (**g**,**h**) are now also classified correctly. Figure shows the improvement achieved with new algorithm in classification of cracks compared to Figure 3.

The results of the deep learning model are trained on the augmented Mendeley Concrete Crack Images for Classification data set (see Section 2.3), and then tested on a subset of test images. Testing of sample images taken in different scenarios are shown in Figure 7. Images without shadows are shown in Figure 7a–d. The accuracy of the model is perfect for both situations—images with and without cracks. The important factor is that the introduction of shadows in the training data set does not result in lower accuracy, and results are comparable with tests shown in Figure 3. Images with shadows and without cracks are shown in Figure 7e,f. Those images demonstrate that False-Positive cases of shadow patterns (that were incorrectly identified as cracks in Figure 3) are no longer present. Finally, images with shadows and cracks are shown in Figure 7g,h. Images with large shadow areas of high intensity are now identified correctly. This demonstrates that the classification using a network trained on the augmented images containing concrete surfaces with shadows results in accurate classification.

**Figure 8:**
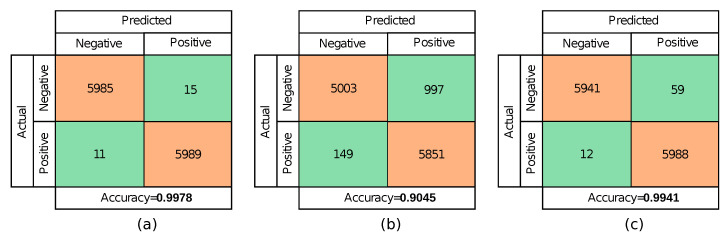
Confusion matrices showing different network accuracy when: (**a**) the model is trained on the original Mendeley “Concrete Crack Images for Classification” data set and tested on concrete images without shadows; (**b**) the model is tested on concrete images with shadows; (**c**) the model is trained on the augmented Mendeley “Concrete Crack Images for Classification” data set and tested on concrete images without shadows.

Confusion matrices showing different network accuracy in different scenarios are shown in Figure 8. First, a confusion matrix for a model trained and tested on the data sets without shadows is given in Figure 8a. The model achieves 0.9978 accuracy and is comparable with most state-of-the-art deep learning-based concrete crack identification models. Test results show 15 False-Positive and 11 False-Negative errors, which is totally acceptable for most applications. Next, a confusion matrix for a model which is trained on the original Mendeley Concrete Crack Images for Classification data set (same as in the previous case) but tested on concrete crack images containing complex shadows of varying intensity is shown in Figure 8b. A noticeable drop in accuracy (0.9045 compared to 0.9978 in the previous case) is observed. The larger part of this drop in accuracy is caused by False-Positive errors. In this case, shadows are incorrectly identified as cracks. Finally, a confusion matrix for a model trained on the augmented Mendeley Concrete Crack Images for Classification data set and tested on concrete crack images containing complex shadows of varying intensity is shown in Figure 8c. In this scenario, the accuracy returns to an acceptable level of 0.9941 and can be considered as a big improvement in comparison to Figure 8b. The presented results prove that the proposed approach based on the augmentation of the original data set with synthetically generated complex shadows proves to be beneficial for real-life applications which require high classification accuracy.

## 4. Discussion

In this paper, we wanted to demonstrate that if machine learning algorithms are used on available databases of concrete crack images, they fail to accurately identify concrete cracks in the presence of challenging environmental conditions. To improve the concrete crack detection accuracy, three options could be proposed: 1st option is to eliminate shadows through pre-processing the acquired images before applying machine learning algorithms for concrete crack detection. This approach does not work well and the drawbacks have been demonstrated in [25]. 2nd option is to improve the concrete crack image database with a large collection of images with shadows in challenging illumination conditions to improve the accuracy of existing deep learning networks, as demonstrated in this paper. 3rd option is to design a completely new deep learning network, which could cater to complex images for challenging environmental conditions, which goes beyond the scope of this manuscript.

There are still some practical challenges that could be envisaged in applying our method using an unmanned areal vehicle (UAV). Most important is to ensure the quality of images acquired by the UAV device. This challenge could be mitigated by using high-resolution imaging cameras, which are readily available these days. Another challenge could be the impact of concrete surface coloring or location of the concrete surface, e.g., onshore or offshore concrete structures (underwater concrete structures). Both of these challenges could be further mitigated with the help of image augmentation. Yet another challenge is with respect to the quantification of concrete crack size. The approach presented in this paper is geared toward concrete crack detection, which is the first step toward detection of concrete crack next step is the quantification of the crack, its size, dimensions, and type of crack. Whether it is a superficial crack, micro-crack, or failure crack. All of them come with their own challenges. Further development of our method is required to address the crack quantification questions. Approach presented in this paper is geared towards preventive maintenance and routine inspections to enable preventive maintenance to ensure that cracks do not lead to failure of the structure.

Although our proposed approach may appear simplistic, it is, however, the most particle approach as it does not require the creation of an entirely new deep learning network and nor does it require massive data collection exercises for images of concrete cracks in challenging conditions. Our approach also demonstrates that its possible to use image augmentation to improve the accuracy of existing established networks, which have been tested on a number of difficult problems.

Finally, the results shown in our paper demonstrated that the augmented data set helped to retrain the network to reach a very high level of accuracy for the classification of images. However, augmentation alone itself should not always necessarily lead to an improved result. The augmentation algorithms introduced in this paper are based on a realistic simulation of optical effects in the virtual digital environment. Therefore, such an approach when an existing data set is augmented and then deep learning algorithms are employed can improve if and only if the augmentation is based on realistic environmental conditions. This has been demonstrated in our paper and this is a major differentiator leading to the success of such an approach.

## 5. Conclusions

This article highlights the challenges associated with the classification of real-life concrete crack images taken in complex illumination conditions. The test experiments conducted on images with shadows clearly demonstrate that current state-of-the-art deep learning models fail to identify cracked surfaces in the presence of shadows. This paper introduces the image augmentation technique, which is achieved through three consecutive steps: ray-tracing of shadows, shadow data set augmentation, and shadow blending. Testing of the model that is trained on the augmented data set shows a significant increase in robustness and the model accuracy—0.9941 compared to 0.9045. The proposed augmentation technique seems to be the most practical approach to help train the networks to identify cracks on concrete surfaces in the presence of shadows as existing data sets can be reused in the process. Such enhanced classification capabilities are beneficial for structural control and health monitoring of concrete structures using unmanned aerial vehicles or drones, where the methodology must be robust enough to deal with real-life images impacted by different environmental conditions such as shadows, shading, blemishes, and concrete spall.

## Figures and Tables

**Table 1:** Architecture of AlexNet deep learning classification network.

Layer No.	Layer Name	Layer No.	Layer Name
1	Image input layer	14	Convolutional layer
2	Convolutional layer	15	ReLU layer
3	ReLU layer	16	Max-pooling layer
4	Cross-channel normalization layer	17	Fully connected layer
5	Max-pooling layer	18	ReLU layer
6	Convolutional layer	19	Dropout layer
7	ReLU layer	20	Fully connected layer
8	Cross-channel normalization layer	21	ReLU layer
9	Max-pooling layer	22	Dropout layer
10	Convolutional layer	23	Fully connected layer
11	ReLU layer	24	Softmax layer
12	Convolutional layer	25	Classification output layer
13	ReLU layer		

## Data Availability

Concrete Crack Images for Classification from Mendeley Data were used in this paper. https://data.mendeley.com/datasets/5y9wdsg2zt/2, accessed on 10 January 2022.

## Abbreviations

The following abbreviations are used in this manuscript:

NN	Neural Network
ANN	Artificial neural network
CNN	Convolutional neural network
R-CNN	Region-based convolutional neural network
LSTM	Long Short-Term Memory
SVM	Support vector machine
KNN	K-nearest neighbors
FCN	Fully convolutional network
UAV	Unmanned aerial vehicle
DSN	Deeply-Supervised Net
